# Topoisomerase IIβ mediates the resistance of glioblastoma stem cells to replication stress-inducing drugs

**DOI:** 10.1186/s12935-016-0339-9

**Published:** 2016-07-26

**Authors:** Saša Kenig, Valentina Faoro, Evgenia Bourkoula, Neža Podergajs, Tamara Ius, Marco Vindigni, Miran Skrap, Tamara Lah, Daniela Cesselli, Paola Storici, Alessandro Vindigni

**Affiliations:** 1 Structural Biology Laboratory, Elettra-Sincrotrone Trieste, Strada Statale 14-km 163, 5, Basovizza, 34149 Trieste, Italy; 2 Department of Medical and Biological Sciences, University of Udine, Piazzale Kolbe 4, 33100 Udine, Italy; 3 Department of Genetic Toxicology and Cancer Biology, National Institute of Biology, Večna pot 111, 1000 Ljubljana, Slovenia; 4 Neurosurgery Unit, Department of Neurosciences, Santa Maria della Misericordia University Hospital, Piazzale Santa Maria della Misericordia, 15, Udine, Italy; 5 Faculty of Chemistry and Chemical Engineering, University of Ljubljana, Večna pot 113, 1000 Ljubljana, Slovenia; 6 Edward A. Doisy Department of Biochemistry and Molecular Biology, Saint Louis University School of Medicine, St. Louis, MO USA

**Keywords:** Glioma, Glioblastoma stem cells, Drug resistance, Replication stress, Theranostic markers, Topoisomerase IIβ

## Abstract

**Background:**

Glioblastoma stem cells (GSC) have been extensively recognized as a plausible cause of glioblastoma resistance to therapy and recurrence resulting in high glioblastoma mortality. Abnormalities in the DNA repair pathways might be responsible for the inability of the currently used chemotherapeutics to eliminate the (GSC) subpopulation.

**Methods:**

In this work, we compared the expression of sixty DNA repair related genes between primary glioblastoma cell cultures and the glioblastoma enriched stem cell primary cultures. MTT test was used to analyze the effect of selected drugs and immunofluorescence to evaluate the load of DNA damage.

**Results:**

We found several differentially expressed genes and we identified topoisomerase IIβ (Top2β) as the gene with highest up-regulation in GSC. Also among the tested cell lines the expression of Top2β was the highest in NCH421k cells, a well-characterized glioblastoma cell line with all the stemness characteristics. On the other hand, Top2β expression markedly decreased upon the induction of differentiation by all trans-retinoic acid. Depletion of Top2β increased the sensitivity of NCH421k cells to replication stress inducing drugs, such as cisplatin, methyl-methanesulfonate, hydrogen peroxide, and temozolomide. Consistently, we found an increased load of DNA damage and increased Chk1 activation upon Top2β depletion in NCH421k cells.

**Conclusion:**

We suggest that Top2β may represent a new target for gene therapy in glioblastoma. In addition, the other genes that we found to be up-regulated in GSC *versus* glioblastoma primary cells should be further investigated as glioblastoma theranostics.

**Electronic supplementary material:**

The online version of this article (doi:10.1186/s12935-016-0339-9) contains supplementary material, which is available to authorized users.

## Background

Glioblastoma (GBM) is the most common and aggressive malignancy of the central nervous system. Despite major progress in cancer treatment in the last decade, GBM remains fatal with 12–15 months median survival after diagnosis [1, 2]. Standard therapy currently consists of minimal resection, followed by radiotherapy alone or in combination with temozolomide. The main cause of mortality in GBM patients is the recurrence of the tumor. According to the cancer stem cell theory, recurrence is a consequence of therapeutics failing to completely eliminate a subpopulation of cancer cells with stem cell characteristic, called cancer stem cells, that have been first described in GBM by Singh et al. [3] and Galli et al. [4]. Besides in GBM [5, 6], the presence of these cells has been confirmed in many tumor types, which has been correlated with worse prognosis in breast, head and neck and oropharyngeal cancer as well as in glioma [7–10]. Several approaches have been suggested to eradicate cancer stem cells, such as induction of differentiation, immunotherapy or genetic manipulation that would block their proliferation or sensitize them to radio- or chemo-therapy [11].

DNA repair pathways were extensively studied in carcinogenesis [12], because defects in these pathways enable tumor cells to accumulate mutations that enhance their proliferation and survival in the complex host tissue microenvironment. At the same time, these DNA repair deficiencies provide the rationale for exploring DNA-damaging drugs for cancer treatment, because treatment with these drugs would cause cell-cycle arrest and consequent cell death in DNA repair defective cancer cells. Following the same reasoning, cancer cells that maintain functional DNA repair pathways might be able to survive chemotherapy and/or radiation. For example, GBM patients are often treated with temozolomide, however the expression of the enzyme O6-Methylguanin-DNA-Methyltransferase MGMT, which removes DNA adducts caused by the DNA alkylating drug temozolomide, makes the drug inefficient in the GBM patient population expressing MGMT. Interestingly, increased resistance of GBM cells to radiotherapy was suggested to be due to DNA damage response preferentially activated in GBM stem cells in comparison to their non-stem cells counterparts [13, 14]. However, the relevance of intrinsic DNA repair efficacy as a resistance mechanism of glioma stem cells remains elusive.

In the present study, we identified a panel of genes that are up-regulated in GSC vs GBM cells, isolated from the same patients. Out of these, we selected topoisomerase IIβ (Top2β) as the most significantly over-expressed in GSC samples. We show that the depletion of Top2β in glioma stem cells leads to increased sensitivity to a panel of replication stress-inducing drugs. This is associated with an increased load of DNA damage, consistent with the previously established role of Top2β in DNA damage repair.

## Methods

### Cell cultures

Glioblastoma (GBM) cell lines U87, U251 and T98G were purchased from American Tissue Cell Culture and grown in DMEM with 10 % FBS in 5 % CO_2_ atmosphere. Glioma stem cell line NCH421k was obtained from CLS cell lines service and grown as floating neurospheres in DMEM/F12 medium supplemented with 0.25 % BSA, 1 % ITS, 20 ng/mL epidermal growth factor (EGF) and 20 ng/mL basic fibroblast growth factor (bFGF). Normal neural stem cells (NSCs, Invitrogen) were grown in KnockOut™ DMEM⁄F-12 Basal Medium, StemPro^®^ Neural Supplement, bFGF and EGF on Geltrex matrix as recommended by supplier (Invitrogen). Normal astrocytes were obtained from ScienCell Research Laboratories and grown in Astrocyte medium (ScienCell Research Laboratories) on poly-l-lysin coated plates.

### Primary cell cultures from human GBM

After surgery tumors were histopathologically reviewed by two independent pathologists and in accordance WHO classification and recommendations. IDH status of glioblastoma tissues was evaluated by Therascreen IDH1/2 RGQ PCR Kit (Qiagen), following manufacturer instructions. All samples used in this study were IDH wild type.

GBM biopsies were mechanically and enzymatically dissociated as described previously [15], and cells less than 40 μm in diameter were cultured both as primary glioblastoma cell culture (GBM) or in a GSC-promoting media. In the first case (primary GBM cell cultures), cells were cultured for 3 days in DMEM supplemented with 10 % FBS and 100 U/ml Penicillin 100 μg/ml Streptomycin at 37 °C and 5 % CO_2_. Alternatively, the cells were grown under the culture conditions promoting selective growth of glioblastoma stem cells (GSC) as neurospheres. Primary GSCs cultures were seeded at a density of 2 × 10^5^ cells in 5 ml of neural proliferation medium composed as follows: Neurobasal-A medium (Gibco), 2 mM l-glutamine (Sigma-Aldrich), 1× N2 supplement (Gibco), 25 μg/ml Insulin, Penicillin–streptomycin, 100 μg/ml human apo-trasferrin (Sigma-Aldrich), 1× B-27 supplement (Gibco), 20 ng/ml h-FGF-basic (Peprotech), 20 ng/ml h-EGF (Peprotech). GSCs were grown as floating neurospheres at 37 °C and 5 % CO_2_. Neurospheres were passaged when they reached approximately 100–150 μm in diameter, by triturating them with a 200 μL micropipette until a homogenous single cell suspension was obtained. Neurospheres were analyzed after minimum three passages.

### PCR-array and RT-PCR

RNA was isolated using Isol-RNA lysis reagent following manufacturer’s instructions (5Prime) and treated with DNase. Quality was examined spectrophotometrically and on agarose gel; 0.5 μg were transcribed to cDNA using cDNA Archive kit (Applied Biosystems). Expression of 60 DNA-repair related genes was measured using custom-made PCR-array (Bar Harbor Biotechnology); full list of genes is presented in the Additional file 1: Table S1. Quantitative RT-PCR reactions were performed on ABI Prism 7900 HT Sequence Detection System using SYBR Green master mix; internal controls were included in the array. PCR conditions were 50 °C for 2 min, 95 °C for 10 min and 45 cycles of 95 °C for 15 s and 60 °C for 1 min; the data were analyzed by the ΔΔCt algorithm.

Expression levels of Top2β and β-actin as endogenous control were measured by PCR (BioRad) using SYBR Green master mix (BioRad) and the following primer pairs (sequences selected from primerdepot NIH): β-actin F: ccttgcacatgccggag, R: gcacagagcctcgcctt; Top2β F: atggccaagtcgggtgg, R: tcatttttgttggcagtttctg; CD133 F: gcattggcatcttctatggtt, R: cgccttgtccttggtagtgt; nestin F: gggagttctcagcctccag R: ggagaaacagggcctacaga. Reaction conditions were the same as for the array.

### Western blot

Cells were lysed in HNNG bufer (20 mM HEPES, pH 7.5, 250 mM NaCl, 0.5 % NP-40, 10 % glycerol, 1 mM phenylmethylsulfonyl fluoride [PMSF]) containing cocktail of protease (Roche) and phosphatase inhibitors (Sigma), 10 μg of proteins from extracts were separated on polyacrylamide gels. Western blotting was performed as previously described using the following antibodies: anti-Top2β (Santa Cruz, 1:500), anti-GFAP (Novus Biologicals, 1:5000), anti β-actin (Sigma, HRP-conjugated, 1:10,000), anti p-Chk1 (Cell Signalling, 1:500), goat-anti mouse HRP-conjugated and goat anti-rabbit HRP-conjugated secondary antibody (Pierce, 1:5000). Western blots were developed with ECL Plus and Image quant LAS4000 imaging system (GE). Quantification was performed using ImageJ software.

### GSC differentiation induction

Differentiation of NCH421k neurospheres was induced as described previously [16] by growing them in the medium as described above, containing 10 % FBS and 10 nM All -Trans-Retinoic Acid (ATRA) for the indicated time.

### Top2β gene silencing

Top2β was downregulated using SmartPOOL RNA mix of 4 siRNAs (Dharmacon, CatN: L-004240-00) and HiPerfect transfection reagent (Qiagen). NCH421k neurospheres or primary GSC spheres were broken to single cell suspension before transfection. Optimal downregulation was achieved by double transfection with 50 nM siRNA and was stable between 48 and 120 h after transfection. RNAi control experiments were performed using siRNA against Luciferase (Dharmacon).

### Cell viability

Cell viability was measured using MTT assay. The drugs used were hydrogen peroxide (H_2_O_2_), temozolomide (TMZ), methyl-methanesulfonate (MMS), cisplatin (CisPt), camptothecin (CPT) and etoposide (all from Sigma). Stock solutions were prepared in DMSO and then diluted in cell media to the final concentrations. Highest final concentration of DMSO was 0.025 % and was confirmed to have no effect on cell survival. Cisplatin (Sigma) stock solution was prepared in physiological solution and diluted to applied final concentration. U251 or NCH421k cells or primary GSC cells were plated on 96-well plates at a density of 2000 cell per well and treated with indicated concentrations of replication stress inducing drugs for 72 h. MTT reagent was added at the final concentration of 5 mg/mL for 3 h. Plates were centrifuged, the violet formazan crystals were dissolved in DMSO and the absorbance was measured at the wavelength 570 nm. Experiments were performed in triplicate (or in duplicate in the case of primary cells), statistical significance was determined by two-tailed Student’s *t* test and p < 0.05 was considered significant.

### Immunofluorescence

NCH421k were transfected with anti-Luciferase siRNA (Luc) or siRNA against Top2β. Spheres were broken down to single cell suspension 72 h after transfection and treated with 50 μM CisPt or 200 μM MMS for 4 h. Cells were then fixed in 3.7 % PFA, permeabilized in 0.5 % Triton X100 and blocked in 3 % BSA. Antibodies used were anti-yH2AX (Santa Cruz, 1:300), 53BP1 (Novagen, 1:500), Alexa Fluor 488 donkey anti-rabbit and Alexa Fluor 596 donkey anti-mouse (Invitrogen, 1:700). Cells were counterstained with Toto3 (1:10,000) and mounted in Vectashield. Images were acquired using Zeiss LSM 510 Meta confocal microscope. Foci were counted with ImageJ ‘Analyze particles’ function. The average number of foci was obtained from three independent experiments analyzing at least 30 cells per sample.

## Results

### Top2β is upregulated in stem cell enriched cultures from primary glioblastoma samples

To test the hypothesis that DNA-repair pathways are involved in the specific resistance of GSC to chemotherapy, we selected 60 DNA-repair genes and studied their expression in primary GBM cell cultures and in the stem cell-enriched populations (primary GSC) obtained from samples histologically confirmed to be GBM. Among these 60 genes, we included genes encoding for factors involved in key double-strand break and single-strand break DNA repair pathways, comprising factors involved in homologous recombination, non-homologous end joining, mismatch repair, nucleotide excision repair and DNA damage response.

We compared seven primary glioma cell cultures with five GSC-enriched primary cultures, out of those pairs three were coming from same patient. Gene expression was normalized to the 18S ribosomal RNA. Based on this analysis, we found 12 genes significantly up-regulated in GSC (Table 1). Among those, Top2β was up-regulated with the highest fold change. Because of the known heterogeneity of GBM tumors [17], we have also compared the three matched samples—primary and GSC-enriched cultures obtained from the same patient. Considering only these sample pairs (Table 2), Top2β was still up-regulated on the average of 2.8-fold with very high significance p = 0.00077. In addition, MSH2 and RPA1 were up-regulated with a p < 0.005. We also performed a blind analysis, where expression is normalized to the genes that are selected with software of the PCR-array producer (Bar Harbor, not shown) and found that Top2β was again on the top of the list with the highest fold increase. Considering all these analysis, Top2β was selected for further studies. Top1 and Top2α were also included in the array, but their expression between primary GBM and GSC enriched cultures was not significantly different (data not shown).

**Table 1:** Expression of DNA repair related genes in primary GBM cultures and GSC-enriched neurospheres

Gene	p value	Fold change
BRCA1	0.011	3.933
PARP2	0.016	3.285
RPA1	0.017	2.569
LIG1	0.018	2.578
CHEK1	0.018	2.854
MSH3	0.020	2.154
XRCC5	0.021	2.559
CHEK2	0.023	2.971
RAD51	0.024	2.260
TOP2B	0.027	4.195
MSH2	0.035	2.250
XRCC1	0.043	2.324

p value and fold change of the genes that were significantly up-regulated in GSC compared to primary GBM cultures

**Table 2:** Expression in GBM and GSC samples originating from the same patient

Gene	p value	Fold change
MSH2	0.0004	2.760
TOP2B	0.0008	2.844
RPA1	0.0013	4.304
MSH3	0.0113	3.240
CHEK2	0.0116	4.080
LIG1	0.0122	5.817
XRCC5	0.0193	3.491
PARP2	0.0308	4.603
RAD51	0.0388	3.733
CHEK1	0.0472	2.539

p value and fold change for the expression of DNA repair related genes in GBM and GSC, when only matched samples were considered for analysis

Expression of Top2β was further validated by RT-PCR on five primary cultures of GBM and GSC of the same patient. The results confirmed the significant 1.8 ± 0.4-fold (p < 0.01) up-regulation of Top2β in GSC with respect to primary GBM cells.

### Expression of Top2β in glioblastoma cell lines, normal neural stem cells and normal astrocytes

To further confirm that Top2β is selectively expressed in GSC, we analyzed its expression levels in different GBM cell lines such as U87, U251, and T98G as well as glioma stem cells NCH421k [both undifferentiated (abbreviated as NCH) and differentiated with all-trans retinoic acid (NCH-A)], normal neural stem cells (NSC), and normal human astrocytes. The latter two cell types are possibly present in GBM parenchyma and in the tumor microenvironment.

**Fig. 1:**
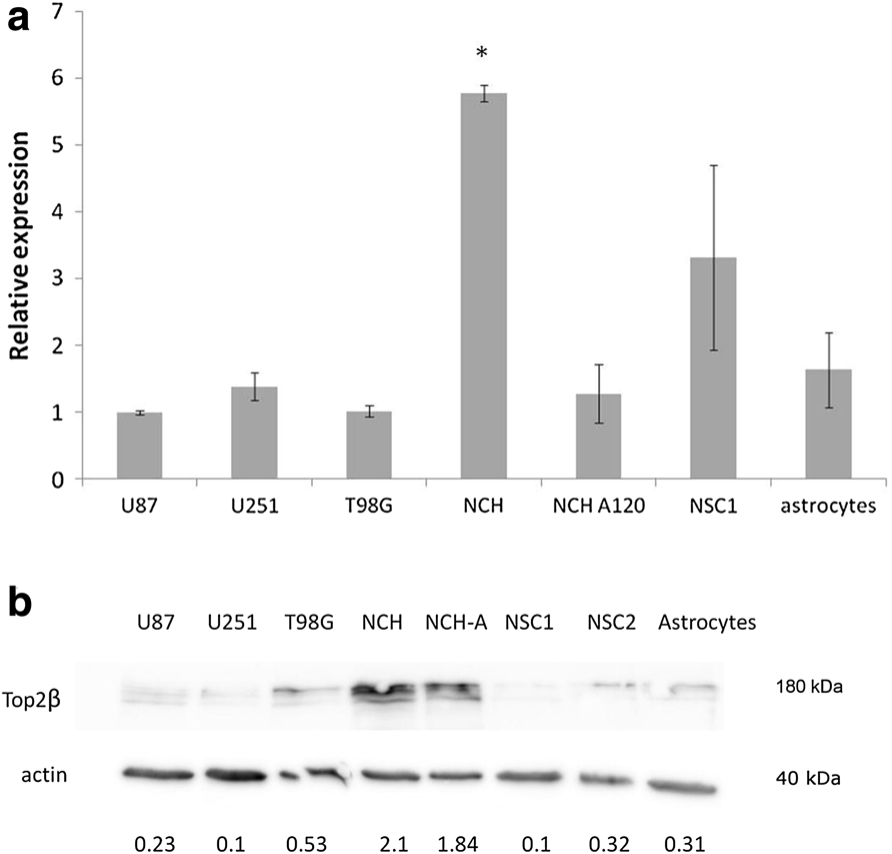
Expression of Top2β in different cell types. **a** Expression of Top2β at mRNA level in established GBM cell lines U87, U251, and T98G, established GSC cell line NCH421k (i.e. NCH) and ATRA-differentiated NCH421k (i.e. NCH-A), normal human neural stem cells (NSC) and normal human astrocytes, measured by RT-PCR, expression normalized to β-actin, average ± SD is presented. **b** Top2β protein expression in established GBM and GSC cell lines as above, as determined using Western Blotting, where β-actin was used as loading control. The numbers below represent the quantitative expression of Top2β normalized to β-actin

We confirmed that the expression of Top2β mRNA level was significantly higher in NCH421k cells than in all other tested cells (Fig. 1a). Specifically, Top2β expression was 5.2 ± 0.9-fold higher compared to GBM cells and 1.8 ± 0.2-fold higher compared to normal neural stem cells (Fig. 1a). Also, when we treated GSC with ATRA, which induces differentiation of GSC towards astrocytes (NCH-A), we found that the expression of Top2β decreased significantly to mean of 22.1 ± 8 % of the original initial value. The expression of Top2β at protein level mirrored the results obtained at the mRNA level, being the highest in NCH421k cells (Fig. 1b).

**Fig. 2:**
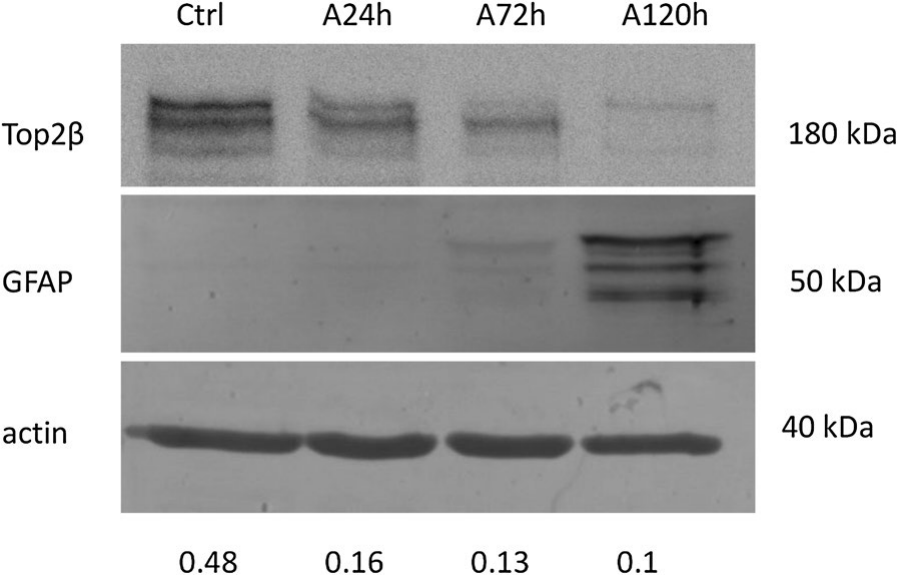
Correlation of Top2β expression and differentiation. The expression of Top2β in NCH421k cells was measured at the indicated time points after ATRA-treatment; GFAP was used as a marker of differentiation, β-actin was used as a loading control. Below the quantification of Top2β expression normalized to actin

To confirm that Top2β expression is related to GBM stemness, we induced GSC differentiation by exposing NCH421k cells to ATRA. We measured the progress of differentiation by the increasing expression of GFAP up to 120 h and found that simultaneously, the expression of Top2β progressively decreased (Fig. 2). Together with GFAP increase we detected also decrease in the expression of stemness markers CD133 and nestin [18]. In the opposite experiment, we down-regulated Top2β expression in NCH421k cells using siRNA to test whether its’ down-regulation alone induces differentiation. However, the expression of GFAP was not detected even after 5 days of Top2β depletion neither did Top2β depletion induce changes in expression of CD133 and nestin. In addition, the expression of GFAP was similar in control and Top2β depleted NCH421k cells both at 24 and 72 h after ATRA-induction of differentiation (data not shown).

### Top2β in glioma stem cells plays a role in DNA damage repair

To study the function of Top2β in GSC, we selected two cell types: the GBM U251 cells and GSC NCH421k cells, expressing the lowest and the highest Top2β levels, respectively. We selected a panel of replication stress inducing drugs, namely camptothecin (CPT) and etoposide that are inhibitors of the topoisomerase 1 (Top1) and topoisomerase 2 (Top2), respectively. CPT primarily causes single strand break formation whereas etoposide induces double-strand break accumulation. We used also the cross-linking agent cisplatin (CisPt), the alkylating agents temozolomide and methyl methanesulfonate (MMS) that form different kind of DNA adducts, as well as hydrogen peroxide (H_2_O_2_) that induces both single and double strand breaks.

**Fig. 3:**
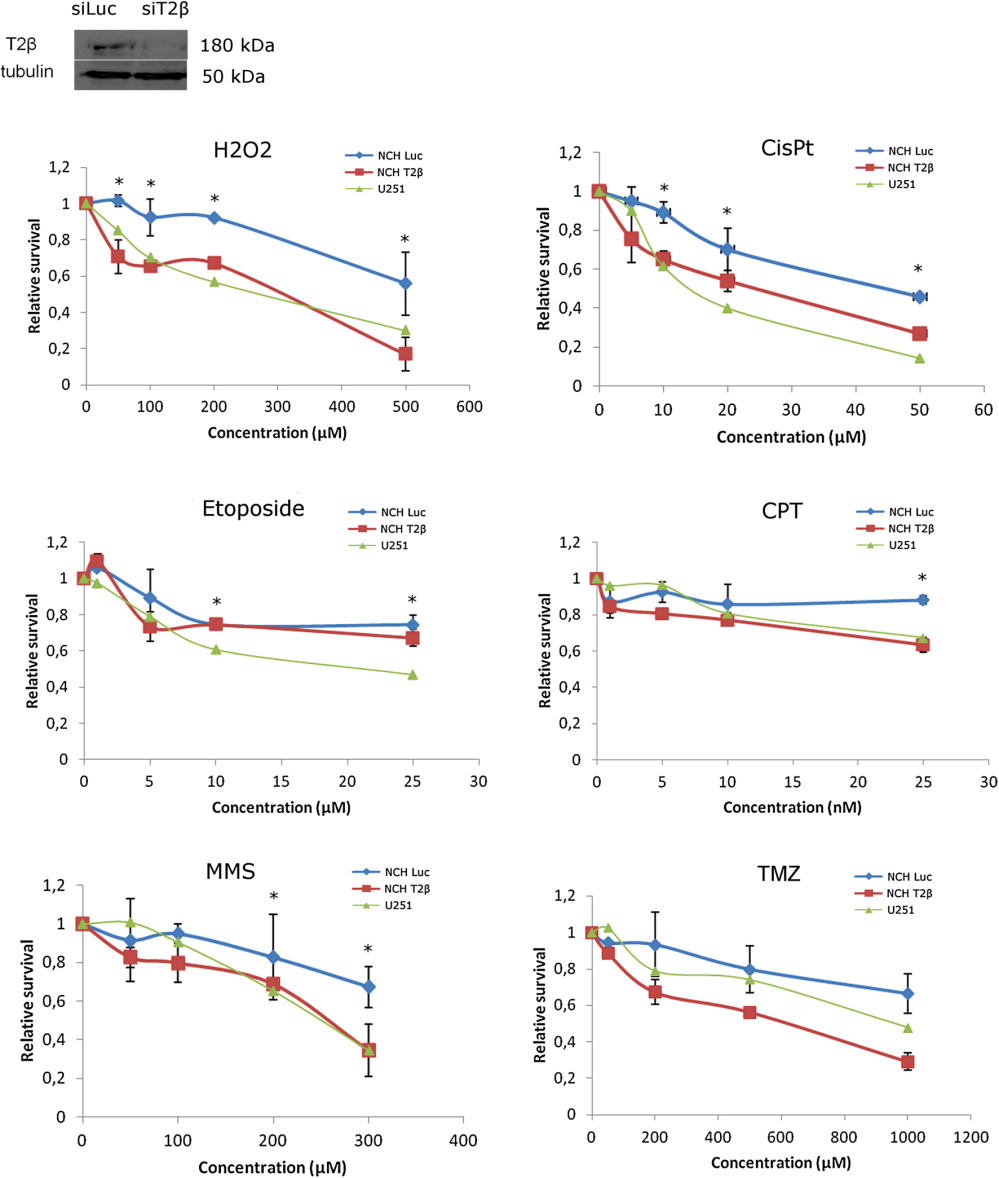
Top2β level dependent sensitivity to replication stress inducing drugs. Survival of U251 and NCH421k cells after exposure to increasing concentrations of selected drugs was determined by MTT assay as described in “Methods”. Drugs used were H_2_O_2_, CisPt, etoposide, CPT, MMS and TMZ. *Green triangles* U251 cells, *blue diamond* NCH421k control cells (siLuc), *red squares* NCH421k with Top2β down-regulation; average of three independent experiments ± SD is presented, *asterisk* indicates significance at p < 0.05. In the inset WB showing the efficiency of Top2β depletion in NCH421k cells

**Fig. 4:**
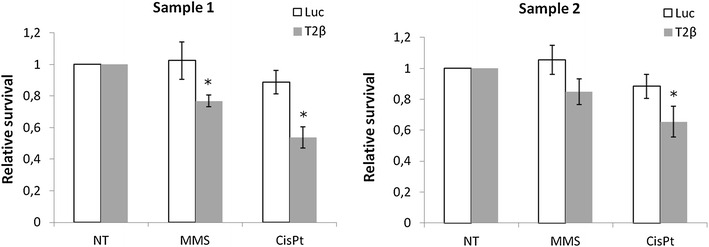
Sensitivity of primary GSC to MMS and CisPt. Top2β was depleted in primary GSC cultures from 2 different patients (sample 1 and 2). Cells were treated with 200 µM MMS or 50 µM CisPt and survival was determined by MTT assay. Average of 2 independent experiments ± SD is presented

We used MTT assays to compare the sensitivity of U251 and NCH421k cells to the selected drugs (Fig. 3). We found that U251 were more sensitive than NCH421k to hydrogen peroxide and CisPt within the entire concentration range, as well as to MMS, etoposide, and CPT at the indicated concentrations. This difference in the sensitivity disappeared upon Top2β depletion in NCH421k cells. Indeed, Top2β depletion (efficient depletion presented in the inset of Fig. 3) sensitized NCH421k cells to MMS, hydrogen peroxide, CisPt and CPT to sensitivity levels comparable to U251 cells and even further in the case of temozolomide. On the other hand, Top2β depletion had no significant effect on the sensitivity to etoposide. Top2β was also downregulated in two primary GSC samples, which caused increased sensitivity to 200 µM MMS and 50 µM CisPt (Fig. 4).

### Increased DNA damage loading

**Fig. 5:**
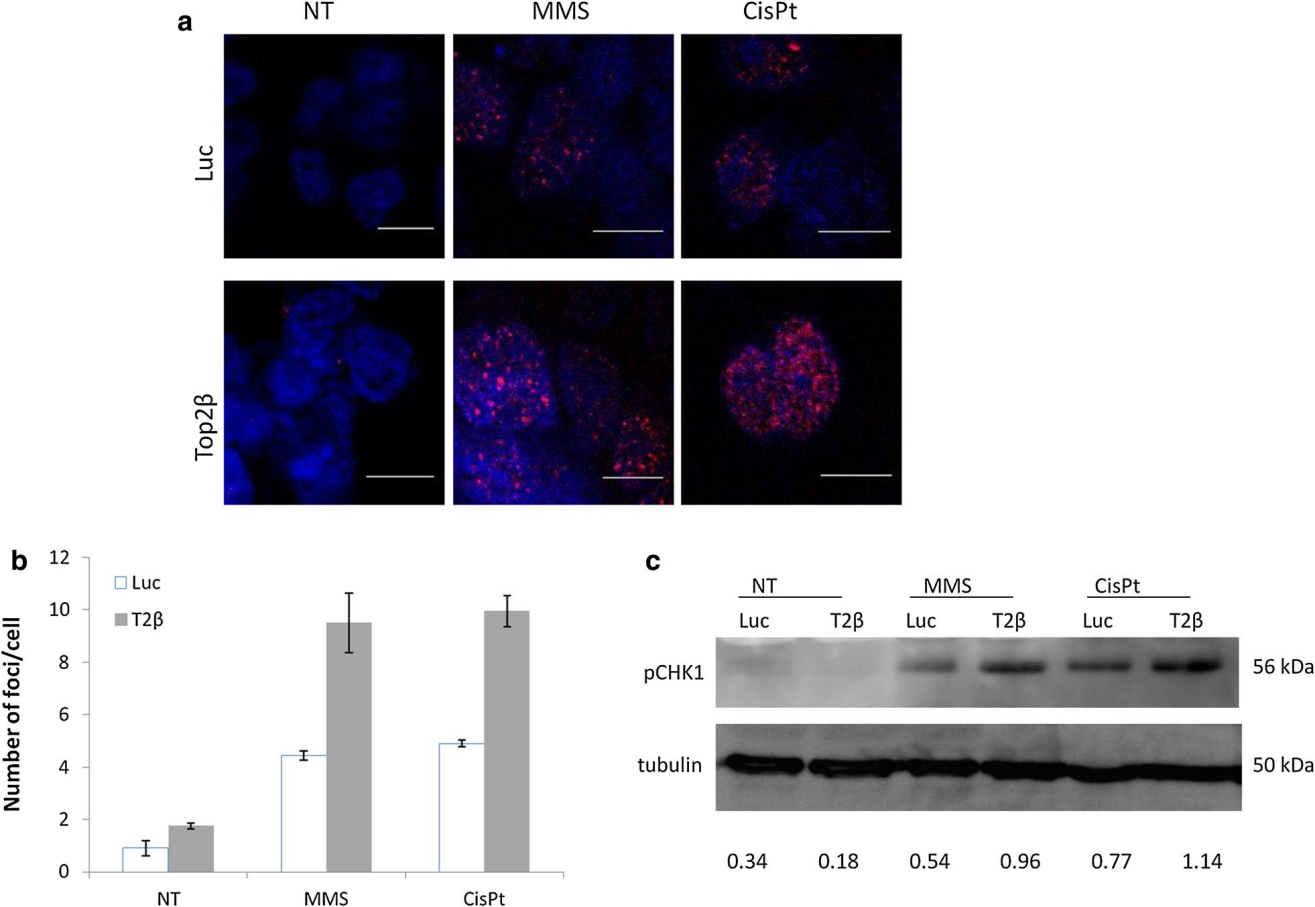
Increased load of DNA damage in Top2β depleted cells. **a** DNA damage was induced using 200 μM methyl-methanesulphonate (MMS) or 50 μM cisplatin (CisPt) in control (Luc) and Top2β down-regulated NCH421k cells. Cells were stained for yH2AX (*red*) and counterstained with Toto3 (*blue*). Representative images are shown, *scale bar* 10 µm. **b** Number of foci per cell was quantified as described in “Methods” and is presented as the average of three experiments ± sd. **c** Chk1 activation after MMS and CisPt treatment, tubulin is a loading control. Below quantification of pChk1 normalized to tubulin

The increased sensitivity detected in Top2β depleted NCH421k cells could be a consequence of an increased load of DNA damage and less efficient repair of DNA lesions upon genotoxic stress induction. To test this possibility, we have analyzed the extent of spontaneous and induced yH2AX foci in control or Top2β depleted NCH421k cells. DNA damage was induced either using 200 μM MMS or 50 μM CisPt. The sensitivity to both of these drugs at this concentration was significantly different for Top2β depleted and control cells. We found that in non-treated cells there was only a minor and statistically non-significant increase in the number of foci per cell—in fact, almost all cells were negative for yH2AX. However, when we induced DNA damage in Top2β depleted NCH421k cells we found that these cells had a 2-fold increase in the amount of yH2AX foci per cell (Fig. 5a, b). In particular, MMS treatment resulted in the formation of 4.2 ± 0.2 per control cell and 9.5 ± 1.1 foci per Top2β depleted cells. Similarly, the number of foci after CisPt treatment was 10 ± 0.6 in Top2β depleted cells, compared to 4.9 ± 0.6 in control cells. Also, both MMS and CisPt treatment induced stronger Chk1 activation in Top2β depleted cells (Fig. 5c).

## Discussion

In the present study we show that Top2β, an ATP-dependent enzyme that catalyzes topological changes of DNA, is highly expressed in glioblastoma stem cells. Enhanced Top2β expression in GSC was initially detected among sixty DNA-repair related genes using PCR array comparing primary GBM cultures and GSC-enriched cultures. The same was then confirmed by RT-PCR. When we extended the same analysis to normal neural stem cells and astrocytes, possibly present in GBM and its parenchyma, we still found predominant expression of Top2β in GSC. The expression in GSC was also higher compared to the expression in established GBM cell lines. Moreover, when we treated stem cells with all-trans retinoic acid (ATRA) that causes GSC differentiation towards astrocytic phenotype, we found that Top2β expression was significantly decreased, strongly suggesting that its increased expression was related to the stemness of GSC.

Top2β is expressed at a basal level in most cells and tissues, including fully-differentiated cerebellum, myometrium and pancreas, or tissues with high cell turnover, such as the endometrium, skin, and bowel mucosa [19]. It has also been shown that the levels of Top2β do not vary much during the cell cycle, even though the protein seems to be differentially phosphorylated during the different cell cycle phases [20]. Interestingly, Top2β expression was also found to decrease with aging, in fact it was almost absent in senescent cerebral granule neurons [21]. Reports on the level of Top2β expression in cancer are very limited. The enzyme was shown to be increased in locally advanced prostate cancer [22]. Top2β expression in GBM and/or in GSC has not been thoroughly investigated so far. However, the publically available Repository for Molecular Brain Neoplasia Data (Rembrandt) (http://caintegrator.nci.nih.gov/rembrandt/) evidences the expression of Top2β in glioma, although it does not correlate with increasing glioma malignancy. Our finding demonstrated higher expression of Top2β in GSCs which are reportedly more abundant in high grade glioma [23] and correlate with worse prognosis [24]. Therefore, we posit that there should be a positive correlation of Top2β with malignancy, but future measurements of the expression of Top2β in low grade glioma would be necessary to support this conclusion.

Following the idea that GBM stem cells are more resistant to chemotherapeutics because of their increased efficiency of DNA repair, we investigated whether increased expression of Top2β is one of the underlying reasons for this phenomenon. First, we have compared the sensitivity of cells with endogenously low (GBM cells U251) and high (GSC cells NCH421k) Top2β expression to selected DNA damage inducing drugs. We indeed found that U251 cells were more sensitive to CisPt, MMS, etoposide and H_2_O_2_ compared to NCH421k cells. Next, we confirmed that down-regulation of Top2β in NCH421k cells increases their sensitivity to these drugs to the same level as detected in U251 cells. Similar increase in sensitivity to MMS and CisPt was observed in the two samples of primary GSC. To determine why Top2β deficient cells are more sensitive to drug treatment, we have also looked at the level of DNA damage by monitoring the phosphorylated form of histone 2AX (yH2AX), which is a hallmark of DNA breaks/damage. In both MMS and CisPt treatments, we found that the level of DNA damage was increased. Also, the checkpoint kinase 1 (CHK1) was increasingly activated upon downregulation of Top2β. Results are consistent with previous findings showing increased sensitivity to H_2_O_2_ in astrocytoma cell line [25] and to N-ethyl N-nitroso urea in granule neurons [26] after Top2β silencing. Moreover, Emmons et al. [27] showed the crosslinking agent melphalan induces an increased level of crosslinks, which lead to apoptosis. Increased sensitivity to genotoxic agent, together with increased accumulation of DNA damage was detected also in ovarian granulosa cells depleted for Top2β [28]. Interestingly, Top2β levels had no noticeable effect on sensitivity to etoposide. This could be a consequence of a partial (but not complete) overlap of the Top2β function with the function of Top2α. Top2α is a direct target of etoposide and cells lacking this enzyme are resistant to etoposide treatment. The hypothesis that Top2β might have a partially overlapping function with Top2α is supported by previous reports showing that the two isoforms of Top2 (170 kDa alpha and 180 kDa beta) have similar enzymatic properties, although they differ in their in vivo expression patterns [20]. Top2β might also have specific functions distinct from those of Top2α consistent with previous reports, showing that etoposide cytotoxicity depends on Top2α, whereas genotoxicity (chromosomal breaks and rearrangements) is Top2β dependent [29]. Also, alkylating agents induced increased levels of cross-links in Top2β down-regulated cells, but not in Top2α down-regulated cells [27]. Of note, we did not detect any significant changes in the expression of other topoisomerases, including Top1 or Top2α. These results suggest that the observed increase in Top2β expression is not a compensatory response connected to deficiencies in other topoisomerase enzymes, as was previously discussed in some contradictory studies [30, 31].

Top2β plays a major role in quiescent (non-S-phase) mouse embryonic fibroblasts exposed to high (μM) CPT concentrations [32]. This was explained with the inability of Top2β depleted cells to replace RNA polymerase II large subunit that is degraded after CPT treatment. In our study, we did not find a major increase in sensitivity of Top2β downregulated cells to CPT. A possible explanation is that we have used low concentrations of drugs with the idea to focus on concentrations that would be potentially useful also under physiological conditions. In case of CPT, we have used concentrations up to 25 nM that have an effect only on replicating cells but not on postmitotic (G1 or quiescent) cells [33] and therefore do not induce significant cell death.

Top2β has also been implicated in cell differentiation. This is particularly important during neuronal development, where Top2β induces the transcription of differentiation related genes through modulation of chromatin structure [34, 35]. Specifically, the signal-dependent activation of gene transcription by nuclear receptors and other classes of DNA binding transcription factors requires Top2β-dependent, transient, site-specific dsDNA break formation [36]. Also, Top2β knockout mice have impaired expression of genes involved in later stages of neuronal differentiation [37]. GSC differentiation was recently suggested as a promising treatment modality, due to impairing the known resistance mechanisms of stem cells. Given that Top2β is over-expressed in cancer stem cells, we also investigated whether Top2β depletion might influence differentiation possibly through modulation of herby relevant genes. However, we found that Top2β down-regulation per se was not sufficient to induce differentiation of GSC. It also neither blocked nor enhanced ATRA-induced differentiation. The effect is likely cell type specific, because Top2β has been shown to repress ATRA induced differentiation of acute promyelocytic leukemia cells towards granulocytes [38].

In addition to Top2β, we also found other genes upregulated in primary cultures of GSC cells, compared to primary GBM cultures. In particular, replication protein A1 (RPA1) and MutS homolog2 (MSH2) were up-regulated with high significance. MSH2 is a protein involved in mismatch repair but its role in GBM drug sensitivity has never been investigated in detail. High levels of MSH2 correlated with glioma malignancy [39] and were detected in multi-resistant malignant gliomas [40]. However, a minor downregulation of MSH2 was found to cause drastic increase in the resistance to temozolomide [41]. RPA1 is a part of replication protein A complex, and has thus far not been studied in GBM. It was however found to have prognostic value in some other types of cancer, such as colon [42]. Further work would be necessary to clarify the function of these additional DNA repair genes in GSC cells. These studies would be important because the proteins coded by these genes might also contribute to the drug-resistant phenotype of GBM.

## Conclusions

Taken together, our results indicate that Top2β is associated with GBM resistance to chemotherapy and supports its stemness characteristics. As such, we posit that Top2β might represent a novel GSC biomarker for diagnosis of therapy resistant tumors and could be used in prediction to GBM therapy response. By the same token, this enzyme can also be target for therapy (so called theranostic), which alone or in combination with other targets, would reduce the GBM stemness, allowing the use of lower doses of chemotherapeutics to eliminate the tumor.

## Additional file

10.1186/s12935-016-0339-9 Full list of genes on the PCR array.

## Abbreviations

GSC: glioma stem cells
GBM: glioblastoma
Top2β: topoisomerase IIbeta
H_2_O_2_: hydrogen peroxide
TMZ: temozolomide
MMS: methyl-methanesulfonate
CisPt: cisplatin
CPT: camptothecin
