# Supplementary material for: Topoisomerase IIβ mediates the resistance of glioblastoma stem cells to replication stress-inducing drugs
Source: Cancer Cell Int. 2016 Jul 26;16:58. doi: 10.1186/s12935-016-0339-9 (PMC4960855; doi:10.1186/s12935-016-0339-9)
Supplement: Supplementary file 1 — 10.1186/s12935-016-0339-9 Full list of genes on the PCR array. [file 12935_2016_339_MOESM1_ESM.docx]

**Supplementary file**

Full list of genes on the PCR array:

| RECQL | RECQL4 | RECQL5 | BLM |
| --- | --- | --- | --- |
| WRN | ATM | ATR | BRCA1 |
| BRCA2 | PARP1 | PARP2 | XRCC1 |
| XRCC2 | XRCC3 | XRCC4 | XRCC5 |
| XRCC6 | PRKDC | MUS81 | EXO1 |
| LIG1 | LIG3 | LIG4 | MRE11A |
| MSH2 | MSH3 | MSH4 | MSH6 |
| MSH5 | POLB | POLD3 | RAD50 |
| RAD51 | RAD51C | RAD51L1 | RAD51L3 |
| RPA1 | RAD52 | TOP1 | TOP2A |
| TOP2B | TOP3A | TOP3B | SMARCAL1 |
| PCNA | TP53 | TP53BP1 | RAD9A |
| RAD1 | HUS1 | CHEK1 | CHEK2 |
| ERCC1 | ERCC2 | ERCC3 | ERCC4 |
| ERCC5 | ERCC8 | NBN | MGMT |
